# Sex-specific differences in plasma lipid profiles are associated with Gulf War Illness

**DOI:** 10.1186/s12967-022-03272-3

**Published:** 2022-02-05

**Authors:** Sarah Oberlin, Aurore Nkiliza, Megan Parks, James E. Evans, Nancy Klimas, Andrew P. Keegan, Kimberly Sullivan, Maxine H. Krengel, Michael Mullan, Fiona Crawford, Laila Abdullah

**Affiliations:** 1 The Roskamp Institute, 2040 Whitfield Avenue, Sarasota, FL 34243 USA; 2 James A. Haley Veterans’ Hospital, Tampa, FL USA; 3 NOVA Southeastern University, Ft. Lauderdale, FL USA; 4 Miami VAMC, Miami, FL USA; 5 Department of Environmental Health, Boston University School of Public Health, Boston, MA USA; 6 Research Service, VA Boston Healthcare System, Boston, MA USA; 7 Department of Neurology, Boston University School of Medicine, Boston, MA USA; 8 Open University, Milton Keynes, UK

**Keywords:** Gulf War Illness, Neutral lipids, Biomarkers, Mass spectrometry, Sex-specific effect, Plasma

## Abstract

**Background:**

Nearly 250,000 veterans from the 1990–1991 Gulf War have Gulf War Illness (GWI), a condition with heterogeneous pathobiology that remains difficult to diagnose. As such, availability of blood biomarkers that reflect the underlying biology of GWI would help clinicians provide appropriate care to ill veterans. In this study, we measured blood lipids to examine the influence of sex on the association between blood lipids and GWI diagnosis.

**Methods:**

Plasma lipid extracts from GWI (n = 100) and control (n = 45) participants were subjected to reversed-phase nano-flow liquid chromatography-mass spectrometry analysis.

**Results:**

An influence of sex and GWI case status on plasma neutral lipid and phospholipid species was observed. Among male participants, triglycerides, diglycerides, and phosphatidylcholines were increased while cholesterol esters were decreased in GWI cases compared to controls. In female participants, ceramides were increased in GWI cases compared to controls. Among male participants, unsaturated triglycerides, phosphatidylcholine and diglycerides were increased while unsaturated cholesterol esters were lower in GWI cases compared to controls. The ratio of arachidonic acid- to docosahexaenoic acid-containing triglyceride species was increased in female and male GWI cases as compared to their sex-matched controls.

**Conclusion:**

Differential modulation of neutral lipids and ratios of arachidonic acid to docosahexaenoic acid in male veterans with GWI suggest metabolic dysfunction and inflammation. Increases in ceramides among female veterans with GWI also suggest activation of inflammatory pathways. Future research should characterize how these lipids and their associated pathways relate to GWI pathology to identify biomarkers of the disorder.

**Supplementary Information:**

The online version contains supplementary material available at 10.1186/s12967-022-03272-3.

## Introduction

Approximately 30% of the 700,000 veterans of the 1991 Gulf War (GW) suffer from Gulf War Illness (GWI), a chronic multi-symptom condition characterized by cognitive impairment, fatigue, and pain [1]. Veterans’ exposure to chemical warfare agents, pesticides, and pyridostigmine bromide are among the major contributors to GWI etiology [1–4]. Currently, there are no biomarkers for indicating the underlying pathology of this condition. Advances in GWI research suggest lipid dysfunction, bioenergetics deficits, and inflammation as key contributors to its ongoing pathology [5, 6]. Blood biomarkers of these biological processes would be a cost-effective and minimally invasive avenue to assist clinicians with accurate detection of GWI.

Evidence suggests that GW pesticides affect cellular lipid metabolism and lipid homeostasis [2, 7–11]. Altered lipid homeostasis can result in an abnormal accumulation of lipids that promote cellular inflammation [12, 13]. Abnormal plasma and brain lipid profiles after GW pesticide exposure in rodents and in veterans with GWI have been shown to accompany inflammation, neurobehavioral and bioenergetic deficits [14–18]. As such, altered plasma lipid profiles may provide a biomarker signature for the bioenergetic deficits and inflammation associated with GWI.

It is expected that since the pesticides to which GW veterans were exposed tend to disturb lipid homeostasis, owing to their high affinity for lipids, neutral lipids classes, such as triglycerides (TG), diglycerides (DG), and cholesterol esters (CE), and phospholipid (PL) classes would be affected in GWI [2, 9–11, 19, 20]. The degree of unsaturation of the fatty acids within the PL classes is known to alter metabolic and inflammatory systems, with many saturated fatty acid (SFA)-containing PL positively associated with chronic metabolic conditions whereas polyunsaturated fatty acid (PUFA)-containing PL are associated with inflammation [21–24]. Among these, PUFA containing the omega-3 fatty acid, docosahexaenoic acid (DHA), generate anti-inflammatory or inflammation-resolving lipid metabolites and PUFA containing the omega-6 fatty acid, arachidonic acid (AA), can produce metabolites that promote pro-inflammatory responses [23–25].

Changes in lipid profiles were also expected in relation to the sex of GW veterans, as many classes of plasma lipids are significantly associated with sex [26]. Women are up to four times more likely than men to be diagnosed with myalgic encephalomyelitis/chronic fatigue syndrome (ME/CFS), a chronic multi-symptom disease clinically similar to GWI that has been shown to have sex-linked differences in blood lipid profiles [27, 28]. Female GW veterans have a higher prevalence of GWI diagnosis and severe GWI-related symptoms than male GW veterans [29]. These findings suggest potential sex-linked differences in pathophysiological responses to GW-era chemical exposures, necessitating sex-stratified analyses.

We hypothesized that blood lipid levels would be affected both by GWI status and the sex of GW veterans. We evaluated the levels of several major blood lipid classes and their molecular species in three cross-sectional cohorts of participants classified as GWI or control. This study will further our understanding of plasma lipids as potential biological markers to track the ongoing inflammation and metabolic dysfunction of GWI, which may help to provide better care and management of this illness.

## Materials and methods

### Cohort characteristics

All protocols were conducted in compliance with the relevant guidelines and regulations and were approved by institutional review boards (IRB) at each institution. Informed consent was obtained from all participants. All plasma utilized within this study were from the following case–control veteran cohorts: (1) the Roskamp Neurology Clinic (RNC) cohort (n = 36), (2) the Boston Gulf War Illness Consortium (GWIC), and (3) the Fort Devens cohort (n = 58) [3, 30, 31]. Additional healthy control samples were provided by Dr. Klimas to supplement the control population of the study. GW veteran controls and the healthy civilian controls were grouped together for analysis in the GWIC cohort (n = 51). In all cohorts, either the Center for Disease Control (CDC) chronic multi-symptom illness definition or the Kansas GWI criteria were used to diagnose GWI [32, 33]. These criteria required that GW veterans must show symptoms in at least 3 of 6 symptom domains (fatigue/sleep problems, somatic pain, neurological/cognitive/mood symptoms, gastrointestinal symptoms, respiratory symptoms, and skin abnormalities) for GWI diagnosis. Exclusionary criteria have been described previously, primarily that participants were excluded if they reported prior diagnosis with a confounding medical condition [17, 30, 31]. Controls were either veterans deployed to the 1991 GW or healthy civilian controls, who did not meet the Kansas GWI criteria or any of the exclusionary criteria. Additional information about the self-report of cardiovascular health status was collected on RNC and GWIC participants. Self-report of any heart conditions, diabetes, high blood pressure or high cholesterol was combined into a single variable to assess cardiovascular risk factors as a group. Reporting of any one or more of these risk factors was coded as presence and a lack of reporting on any cardiovascular risk factors was coded as absence.

Data on the sex of the participants were gathered via self-report, with the options being either male or female. As per the National Institute of Health, sex refers to biological differences between males and females [34]. As per the World Health Organization, gender refers to the characteristics of men and women that are socially constructed [35]. Therefore, this paper evaluating biological lipid levels uses exclusively male/female terminology for participants rather than men/women terminology, as data on the gender of the participants was not collected.

Participant recruitment, blood collection, and blood storage procedures have been described previously [17, 18, 31]. All samples were collected using the same written standard operating procedures for performing phlebotomy, plasma separation and aliquoting. All participants had fasted prior to blood collection. There was no requirement of time of collection.

Due to low numbers in ethnic groups other than Caucasian, we combined participants who were African American, Aleutian Eskimo, American Indian, Asian, Pacific Islander, multiracial or other races into one category, called Non-Caucasian. Only one African American veteran with GWI was present in the RNC cohort. Of the eleven African American participants in GWIC, three were control participants and eight were veterans diagnosed with GWI. Of the six African American participants in the Fort Devens cohort, four were control participants and two were veterans diagnosed with GWI.

### Lipidomic analysis and quantification

Plasma samples were randomized prior to use via a group-block design, to ensure that the experimenters were blinded to participants’ group membership and other characteristics. The extraction protocol and liquid chromatography–mass spectrometry (LC–MS) run settings have been previously described by our laboratory [18, 36]. Briefly, lipid extracts from 10 µL of plasma spiked with class-specific internal standards (to control for variability in lipid extractions) were resuspended in 200 µL of 70:30 LC–MS mobile phase A:B [36, 37]. Mobile phase A was composed of 42% water, 31% acetonitrile, 27% isopropanol, 10 mM ammonium formate, and 0.1% formic acid. Mobile phase B was composed of 90% isopropanol, 10% acetonitrile, 10 mM ammonium formate, and 0.1% formic acid. For LC–MS analysis, an Easy-nanoLC 1000 chromatography pumping system was used with a nanoflex electrospray ionization (ESI) source on a Thermo LTQ/orbitrap mass spectrometer.

Resuspended lipid extracts (4 µL) were injected onto an Acclaim PepMapTM 100 (75 µm × 2 cm, nanoViper, C18, 3 µm, 100 Å) trapping column attached to an Acclaim PepMapTM RSLC (75 µm × 15 cm, nanoViper, C18, 2 µm, 100 Å) analytical column for reversed-phase chromatographic separation of lipid species. The chromatographic gradient started at 30% B, then increased linearly from 1 to 40 min from 50 to 98% B, plateauing at 98% B from 40 to 50 min, and then decreasing to 30% B for 50 to 65 min. All samples were run in triplicate, accompanied by a blank and a quality control sample in each run. Full-scan mass spectrometry (MS) data was acquired in positive ion mode, over a mass range of 300–2000 m/z at a resolution of 30,000. A maximum inject time of 200 ms was used with 6 microscans/acquired scan. Thermo Xcalibur Qual Browser was used to examine the individual chromatographic data of samples for lipid retention times. Lipid peak areas were integrated using Tracefinder™ software, using target compound lists of analytes of interest, identifying them based on m/z and retention time. A high mass accuracy window of 5 mmu was used for detecting each individual lipid species. We detected several major classes of lipids including the phospholipid classes phosphatidylcholine (PC), phosphatidylethanolamine (PE), the neutral lipid classes TG, DG, and CE and the sphingolipid classes sphingomyelin (SM), ceramides (Cer) and hexosylceramides (HexCer), which were quantified as previously described [28, 36]. The quality control (QC) samples were then used to normalize between batches of data ([sample] x [batch QC/normalizing QC]). Once the coefficients of variance (CV) were calculated per class for the whole cohort, lipid species with an average CV > 25 were excluded from further analysis. Lipid species with less than 90% coverage were also excluded from our analyses.

To categorize lipids based on their degree of unsaturation, totals were calculated by adding up values for all lipid species measured belonging to that lipid class. Saturated fatty acids (SFA) were calculated by totaling the lipids with no double bonds; monounsaturated fatty acids (MUFA) were calculated by totaling the lipids with one double bond. Polyunsaturated fatty acids (PUFA) were calculated by totaling all measured lipid species with three or more double bonds. For lipid classes known to contain AA or DHA, their respective categorizations were made by totaling individual species known to contain the relevant type of fatty acid, as based on previous MS/MS confirmations.

### Statistical analysis

Chi-square and one-way analysis of variance (ANOVA) tests were used, as appropriate, to compare between the three cohorts and to compare GWI and healthy controls for descriptive purposes. Each lipid species was assessed for normality and those found to be skewed were log transformed for parametric analysis. All lipidomics data were analyzed using mixed linear modeling (MLM) as described previously to examine the independent effects of sex and GWI diagnosis as well as to account for random effects associated with repeated measurements in lipidomics and metabolomics datasets [17, 36–39]. The Benjamini-Hochberg (B-H) correction procedure was performed to correct for multiple testing and the false discovery rate (FDR) limit was set at 0.05. The webtool MetaboAnalyst 5.0 was used to perform a hierarchical clustering analysis of the individual lipids with a coverage superior to 90% [40]. Clusters were determined based on the similarity of lipids as expressed by the Euclidean distance. Lipids that were adjacent based on the Ward algorithm were combined in clusters and an ANOVA was applied to select the top 50 lipids significantly modulated by sex and diagnosis represented in the dendrogram and heatmap.

## Results

### Basic demographics

General demographic characteristics of participants from the three cohorts at the time of the blood collection are presented in Table 1. The difference in ages between the RNC participants and the GWIC participants was found to be significantly different (p < 0.01). The Fort Devens and GWIC cohorts were found to be significantly different for race (p < 0.05). Overall, the GWIC cohort was younger and had a higher proportion of non-Caucasian participants than the other two cohorts (Table 1). The percentage of female participants ranged from 13.7% to 27.8% across the 3 cohorts (Table 1).

**Table 1:** Descriptive table of demographic variables of the three cohorts: RNC, GWIC, and Fort Devens

	RNC (n = 36)	GWIC (n = 51)	Fort Devens (n = 58)
	Mean (SD)		
Age**	55.4 (1.2)	50.3 (1.1)	55.9 (1.2)
BMI	29.4 (5.5)	30.8 (4.8)	–
	N (%)		
GWI Cases	31 (86.1)	31 (60.8)	38 (65.5)
Female	10 (27.8)	7 (13.7)	13 (22.4)
Race*			
White/Caucasian	31 (86.1)	34 (66.7)	50 (86.2)
Non-Caucasian	5 (13.9)	17 (33.3)	8 (13.8)
Cardiovascular risk factors	19 (55.9%)	21 (50.0%)	
Diabetes	6 (17.6%)	4 (9.3%)	–

The mean age and standard deviation (SD) are reported. Significant differences are indicated by either single asterisks (p < 0.05) or double asterisks (p < 0.01).

When all cohorts were combined (Table 2), there were no significant differences for age and race between GWI cases and controls. However, the sex ratio was found to be significantly different between GWI cases and controls, as 16% of GWI cases were female compared to 31% of controls (p < 0.05). In a subset of participants, there were no differences between GWI cases and controls for BMI, the presence of diabetes and the presence of cardiovascular risk factors. There were no differences between males and females for the presence of diabetes or cardiovascular risk factors (p > 0.05). Similarly there were no significant differences between males and females for BMI (males: 30.1 ± 5.3 SD and females: 29.4 ± 4.2SD). Given the known associations between sex and blood lipid levels and differences in the proportion of male and female sex between GWI and control participants, subsequent analyses were stratified by sex to account for its confounding effects.

**Table 2:** Descriptive table of demographic variables of GWI cases and controls from the RNC, GWIC, and Fort Devens cohorts combined

	Controls (n = 45)	GWI (n = 100)
	Mean (SD)	
Age	55.6 (10.8)	53.1 (7.1)
BMI	30.1 (3.8)	30.0 (5.4)
	N (%)	
Female*	14 (31.1%)	16 (16.0%)
Race		
White/Caucasian	35 (77.8%)	80 (80.0%)
Non-Caucasian	10 (22.2%)	20 (20.0%)
Cardiovascular risk factors (Yes)	8 (50.0%)	32 (53.3%)
Diabetes	1 (6.3%)	9 (15.0%)

Significant differences are indicated by single asterisks (p < 0.05).

### Sex-specific differences were observed for the degree of unsaturation and AA and DHA content of several major classes of lipids in veterans with GWI

**Fig. 1:**
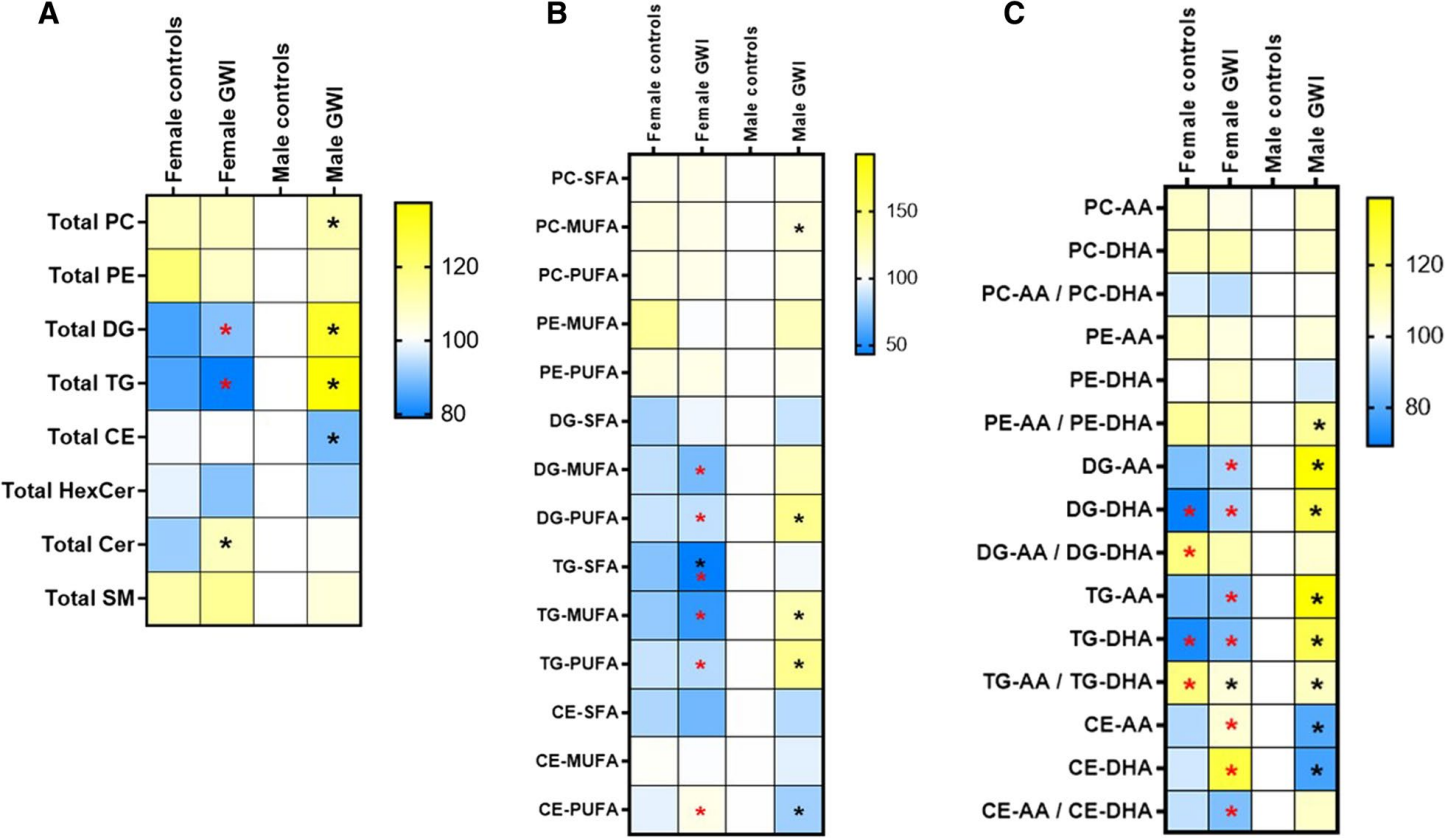
Heatmap visualization of phospholipids and neutral lipids in plasma of GWI cases and controls. The heatmaps represent the average concentrations relative to male controls of the **A** total level of each lipid classes, **B** lipids stratified by their degree of unsaturation and **C** lipids containing arachidonic acid (AA) or docosahexaenoic acid (DHA) as well as their AA-containing lipids/DHA-containing lipids ratio in each group. Black asterisks indicate significant differences between GWI cases and their sex-matched controls (p < 0.05). Red asterisks indicate significant differences between males and females within the same diagnostic group (p < 0.05)

Figure 1 shows heatmaps of plasma profiles of eight different lipid classes that are differentially affected by sex and diagnostic differences among male and female participants (see Additional file 1: Fig. S1 for individual values). The total amount of PC, DG and TG were significantly increased while the total amount of CE was significantly decreased in male GWI compared to male controls (Fig. 1A). Among female participants, only total Cer were significantly increased in GWI compared to controls. We also evaluated the degree of fatty acid unsaturation in individual lipid species since neutral lipids are considered reservoirs of PUFA lipids (Fig. 1B). Compared to male controls, male veterans with GWI had a significant increase in the unsaturated lipids, consisting of DG-PUFA, TG-MUFA, TG-PUFA and PC-MUFA, while CE-PUFA was significantly decreased. Among female participants, TG-SFA levels were significantly lower in GWI compared to controls. The PUFA-containing lipid species were further grouped by their AA or DHA-content for each lipid class (Fig. 1C). Levels of DG-AA, DG-DHA, TG-AA and TG-DHA were significantly increased while CE-AA and CE-DHA were significantly decreased in male GWI cases compared to male controls. The ratios of PE-AA/PE-DHA and TG-AA/TG-DHA were both significantly increased in male GWI compared to male controls. The TG-AA/TG-DHA ratio was also significantly increased in female GWI compared to female controls. Independent effects of the presence of cardiovascular risk factors on these lipid profiles are presented in Additional file 2: Fig. S2.

### Hierarchical clustering analysis identifies sex-specific lipid species signature of GWI

**Fig. 2:**
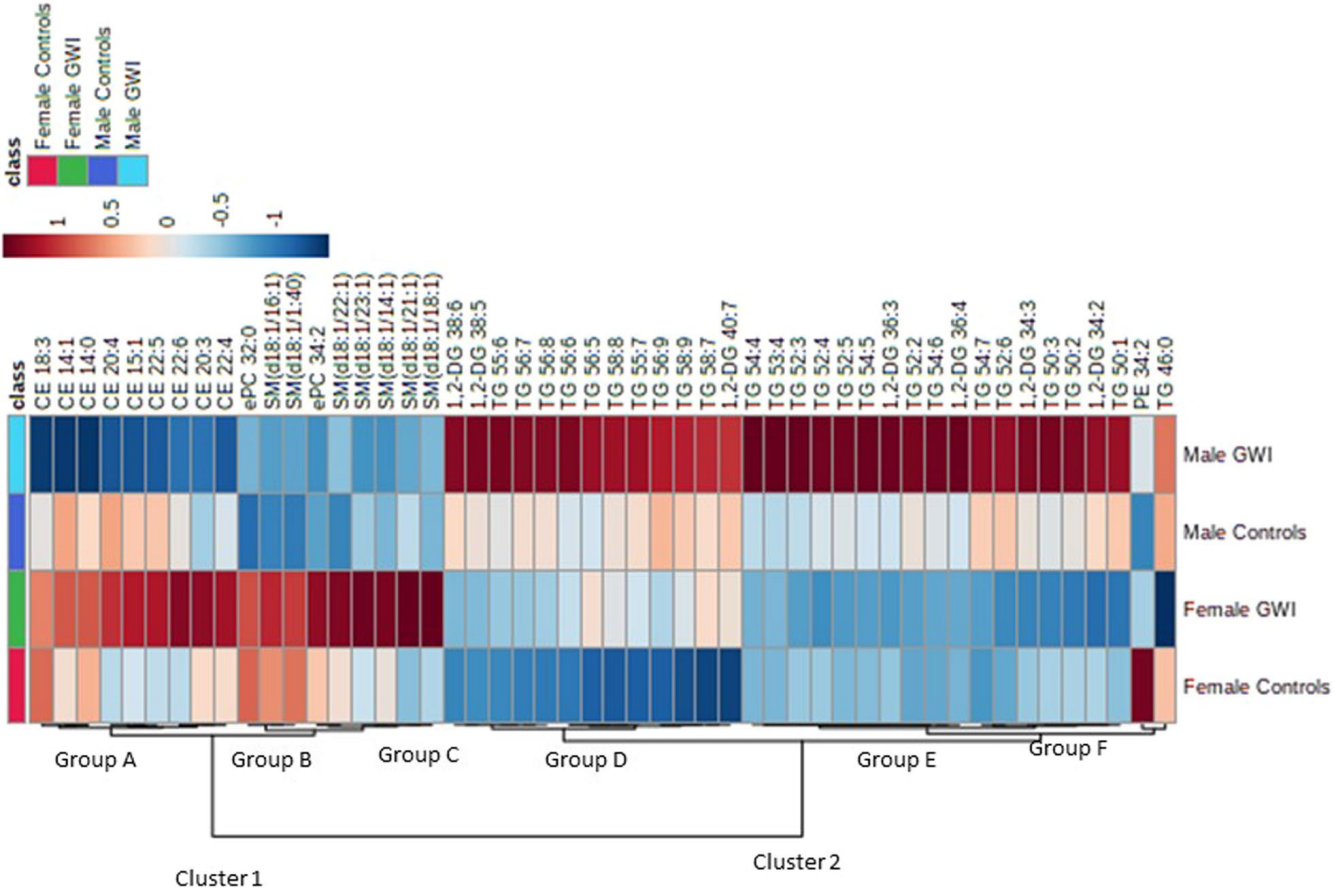
Hierarchical clustering of the top 50 lipids. The relative concentration value of each lipid from the top 50 lipids significantly modulated by sex and diagnosis identified by MetaboAnalyst has been projected onto the heatmap for each group. The color legend identifies in red the lipids with higher concentration and identifies in blue the lipids with lower concentration

Hierarchical clustering analyses identified two major clusters (cluster 1 and cluster 2) within the top 50 lipid species significantly modulated by sex and diagnosis (Fig. 2). Lipids within both clusters were affected by sex differences, irrespective of GWI diagnosis. Cluster 1 was primarily composed of the neutral lipid class CE and the sphingolipid class SM and showed a phenotype of increased levels in female GWI and decreased levels in male GWI. Cluster 2 was primarily composed of the neutral lipid classes DG and TG, which were increased in male participants compared to female participants, irrespective of GWI diagnosis. Lipids associated with each cluster further separated into 3 subgroups. The CEs of group A show controls to be generally similar regardless of sex while the lipids differed in concentration in the GWI veterans based on sex. Lipids associated with groups B and E were both principally influenced by sex regardless of the diagnostic status. Group B was composed of an ether PC and SM lipids, while Group E was composed of DGs and TGs. Lipids in group C differed between female GWI and female controls. In group D, neutral lipids of the male and female GWI groups were both increased compared to their sex-matched controls. Both group D and E were composed of the neutral lipids, DG and TG, and notably differed in their range of degree of unsaturation, with lipids in group D consisting of more double bonds and those in group E consisting of fewer double bonds. Group F consisted of 2 lipid species that are atypical of any other group, but do not match each other either. PE34:2 is highly increased in the female controls and decreased in all other groups, while TG 46:0 is highly decreased in the female GWI group and increased in all other groups.

**Fig. 3:**
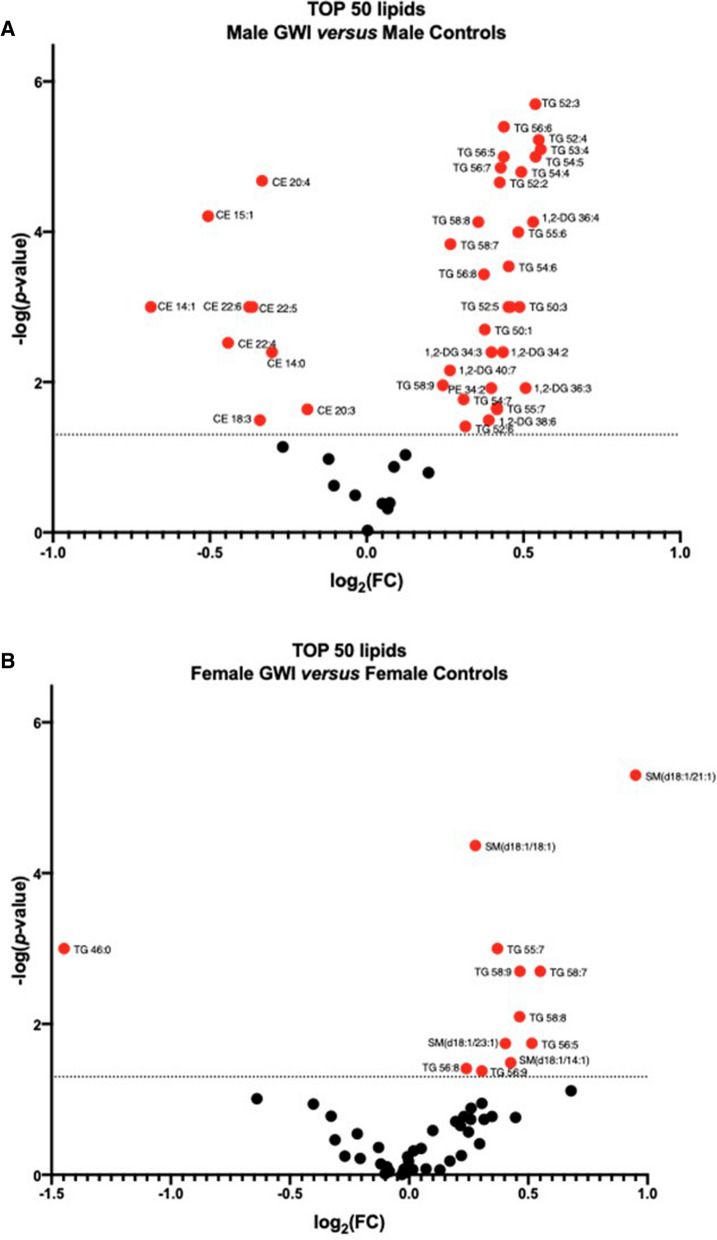
Fold Change of the top 50 lipids by sex. Relative changes of the top 50 lipids significantly modulated by sex and diagnosis in males (**A**) and females (**B**) between GWI participants and GW cases are shown by fold change (FC) on the volcano plots. Each dot represents one individual lipid from the list of top 50 lipids identified by MetaboAnalyst. The red dots indicate the significant changes relative with *p*-value threshold set at 0.05 (horizontal dotted line)

Of the top 50 lipids significantly modulated by sex and diagnosis, more lipid species were significantly modulated in male GWI cases (Fig. 3A) than in female GWI cases (Fig. 3B) as compared to their respective sex-matched controls. Among male participants, TG and DG were significantly increased by 1.18-fold at minimum (TG 58:9, log_2_FC = 0.24) and all CEs were significantly decreased by 0.88-fold at minimum (CE 20:3, log_2_FC = − 0.19) in GWI veterans versus controls. A few TGs and SMs were significantly increased by a minimum of 1.18-fold change (TG 56:8, log_2_FC = 0.24) in female GWI participants compared to female controls, except for TG 46:0, which was significantly decreased by 0.37-fold (log_2_FC = − 1.45).

## Discussion

Previous animal studies have shown that exposure to GW chemicals, such as those to which GW veterans were exposed, disturbs lipid homeostasis and contributes to inflammatory and metabolic dysfunction that are seen in veterans with GWI [7–9, 14, 15, 41]. To evaluate the translational significance of prior mechanistic studies, the current study analyzed plasma lipid profiles of veterans with GWI and control participants. Most of the differences pertained to the neutral lipids (CE, TG and DG), where the degree of unsaturation and ratios of AA-to-DHA were changed in GWI compared to controls. Our study shows that male veterans with GWI had significant increases in total neutral lipid content compared to male controls. Female veterans with GWI had elevated levels of Cer and lower levels of SFA TG species as compared to female controls. These sex-specific plasma lipid compositions in GWI veterans require further attention as they may indicate progressive metabolic and inflammatory processes, particularly in male veterans with GWI.

In the plasma, neutral lipids primarily appear in lipoprotein compartments due to their hydrophobic nature. The neutral lipid class of TG are major dietary fats, but they are also synthesized in the liver [42]. Because dietary TGs are digested to free fatty acids and monoglycerides in the intestines, plasma TG levels within lipoprotein particles are reflective of TG synthesis in liver via acylation with DG [43]. In plasma, an excess of TG, such as seen in the male veterans with GWI, is thought to reflect an overproduction of very low-density lipoprotein (VLDL) corresponding with the production of CE-depleted low-density lipoprotein (LDL) particles [42]. This production of CE-depleted LDL may explain the significant decrease in CE in male veterans with GWI. In the current study, female participants, irrespective of their diagnosis, had lower levels of TG and DG compared to their male counterparts. This has also been reported by the Genetics of Lipid-Lowering Drugs and Diet Network (GOLDN) study of plasma lipidome, who attributed this to normal sex-linked lipid differences [26]. In terms of the clinical implications of elevated TG in male veterans with GWI, elevated plasma TG is thought to be a risk factor for cardiovascular disease [44]. Elevated TG and the generation of CE-depleted LDL particles are also the defining features of dyslipidemia associated with insulin resistance and type 2 diabetes mellitus [42].

Veterans with GWI may be more sedentary and exercise less than healthy controls due to their condition, so this TG increase could reflect heightened body weight in veterans with GWI. A positive association between high TG and elevated body mass index (BMI) has been previously reported [45, 46]. As early as 1995, it was observed that veterans with severe GWI had slightly higher BMI than non-cases [33]. However, BMI did not differ between GWI and controls (in a subsample) and therefore unlikely to explain the observed differences in lipid profiles. A recent health survey of GW veterans from the Fort Devens cohort suggested increases in the prevalence of high blood pressure, diabetes, and cardiovascular disease over the last 30 years [4]. The Fort Devens Cohort was also assessed to have significantly higher rates of diabetes in both male and female GW veterans than the general population [47]. However, differences between GWI and controls were not evaluated in this prior study. In our current study, a lack of difference in cardiovascular risk factors between controls and GWI suggested that observed differences in lipid profiles between the two groups were not simply a reflection of the presence of cardiovascular risk factors in this study cohort. As many of the neutral lipid changes may be indicative of subsequent risk of cardiovascular disease with age, further assessement of blood lipids by clinicians may be helpful for monitoring cardiovascular health as veterans with GWI age.

In the current study, SFA-containing TG species were reduced in female veterans with GWI. The role of SFA in promoting cardiovascular disease remains controversial as the source of these SFA-containing lipids and the individual identity of each SFA influences their potential role in promoting cardiovascular disease [21, 48]. As the SFA-TG species measured ranged from TG 46:0 to TG 54:0, and therefore consisted of 46 to 54 carbons split amongst 3 fatty acid chains, it is likely that the SFA-TG species measured contained long-chain fatty acids, which are over 12 carbons in length [48]. However, their significance in the clinical presentation of female GWI is unclear. Since these same TG-SFA species were elevated among those with cardiovascular risk factors, future studies aimed at different aspects of dietary modification in male vs. female GW veterans may be required to better understand the role of SFA-containing TG species in the health, particularly cardiovascular health, of veterans with GWI.

Our previous studies using subsets of samples from these cohorts showed increases in proinflammatory cytokines, interleukin-1 beta (IL-1β), interferon gamma (IFN-γ) and IL-6, and increases of peripheral immune cells among veterans with GWI compared to controls [18]. Others have shown that veterans with GWI have increases in other blood inflammatory markers consisting of C-reactive proteins and matrix-metalloproteinase-2 [49]. Several proinflammatory cytokines, including IL-1β and IL-15, are associated with an increase in GWI symptom severity [50]. As such, an examination of lipids related to inflammation may provide additional information about the underlying biology of GWI. Since omega-6 AA or omega-3 DHA modulate inflammation and comprise a large proportion of PUFA, we focused on lipids that contain AA and DHA as their fatty acid side chains. Our previous studies have shown that the AA and DHA content within certain PL classes were associated with GWI [17]. We now show that ratios of AA-to-DHA were increased in TG species among male and female veterans with GWI. The ratio of AA-to-DHA was also increased in PE among male GWI veterans. As AA and DHA compose 50% and 40% of brain PUFAs respectively, sufficient AA is necessary for the growth, repair, and maintenance of neurons and DHA is used in neurotransmission [51]. However, AA metabolites, such as prostaglandins, are known to activate pro-inflammatory pathways whereas DHA metabolites, such as resolvins, contribute to inflammation resolution pathways [24, 52]. In addition, studies have shown that patients with a low DHA/AA ratio, (i.e. a higher AA-to-DHA ratio) had a higher risk of acute coronary syndrome than those with a high DHA/AA ratio, and this was significant for men in particular [53]. These differences in AA and DHA composition could reflect a generalized pro-inflammatory state in veterans with GWI, which would be consistent with reports of markers of chronic, low-grade inflammation being associated with GWI diagnosis [49, 54]. Hence, future studies are needed that interrogate systems-level omic data along with biological parameters of inflammation to identify specific inflammatory pathways that are associated with GWI pathogenesis.

Another indication of pro-inflammatory states in GWI would be the increase in Cer in female GWI veterans, as ceramides are known to stimulate the production of pro-inflammatory cytokines, such as IL-6 and TNF-α, in macrophages and are correlated with IL-6 levels in serum [55, 56]. Production of long-chain ceramides is known to induce apoptosis [57]. The increase in the long chain Cer measured in this study (d32:0–44:2) may therefore indicate increased cell death in female GWI veterans. Ceramide levels are shown to be increased with obesity and metabolic disorders, particularly diabetes. For instance, Boon and colleagues also found that Cer elevation is another marker of the dyslipidemia of type 2 diabetes mellitus and promotes insulin resistance [55]. Others have shown that plasma Cer increases are associated with obesity and may be indicative of lipotoxicity, which may ultimately contribute to the development of diabetes and insulin resisistance [58, 59]. These studies suggest a different process in males and females in relation to Cer-mediated metabolic dysfunction that warrants further investigation.

This study provides key information about blood biomarkers associated with adverse metabolic and inflammation-related lipid profiles. The BMI in females was non-significantly lower than in males and therefore did not present as a confounding factors for the differences in lipids seen between the two sex groups. These findings are largely consistent with previous studies showing sex-specific differences in lipid profiles. Another limitation of our study includes unavailability of dietary data and medication data, particularly on the use of statins and omega-3/fish oil supplements. Since statins lower TG levels in blood and our findings suggest increases of TG in male veterans with GWI, we anticipate that it is unlikely that statin use affected our study results. Similarly, we have previously shown that AA-to-DHA ratios are decreased after DHA supplementation [39]. Since we observed an increase in AA-to-DHA ratios in male GWI veterans, it is similarly unlikely that omega-3 or fish oil supplementation was a confounder in our study. As CE levels were lower in male GWI veterans, measuring free cholesterol would be useful in future studies to determine if either the level of free cholesterol or the rate of cholesterol esterification had decreased. Unfortunately, we could not reliably detect the labelled free cholesterol in our assay but will correct this in future studies. While we have previously examined free fatty acids in a smaller cohort of GW veterans, which suggested that free forms of PUFA were indeed affected in GWI [18], a large study evaluating free fatty acids and their bioactive lipid metabolites will help better elucidate the role of these fatty acids in ongoing inflammatory processes associated with GWI.

## Conclusion

This study identifies distinct sex-specific plasma lipid profiles associated with GWI diagnosis, with significantly elevated TG and DG and lowered CE as a distinctive pattern in male GW veterans with GWI. Triglyceride AA-to-DHA ratios were also increased in male GW veterans with GWI. Since these lipid changes are associated with metabolic syndrome and inflammation, our findings suggest veterans with GWI have maladaptive biological processes that involve lipid transport and metabolism. While additional mechanistic work is needed to better elucidate lipid dysfunction in GWI, these blood lipid signatures suggest that focused studies are needed into the role of dyslipidemia in the ongoing pathophysiology of GWI. The current findings suggest that blood lipid monitoring may be beneficial for veterans with GWI. As such, further research is needed to characterize how these lipids and their associated pathways relate to GWI pathology to identify potential biomarkers for tracking general health related outcomes in GWI.

## Supplementary Information

**Additional file 1: Figure S1.** Individual levels differences in major blood classes between GWI and controls. Bar graphs indicate mean ± SE and individual values of total lipids (A), degree of unsaturation of each lipid class (B) and AA and DHA content of these lipids (C) that were significantly different between controls and GWI. Black asterisks (*) indicate significant differences between GWI cases and their sex-matched controls (p < 0.05).

**Additional file 2: Figure S2.** Association of blood lipid profiles and the presence of cardiovascular risk factors among the study population. Major lipids were further evaluated to determine the impact of presence of reported cardiovascular risk factors (no = 8 and yes = 32). Heatmap generated from z-scores transformed from individual concentrations. Differences in AA and DHA containing PL and neutral lipids were affected the presence of cardiovascular risk factors. Total lipid content was unaffected by cardiovascular risk factors. Black asterisks (*) indicate significant differences between GWI cases and their sex-matched controls (p < 0.05).

## Data Availability

The raw data supporting the conclusions of this article will be made available by the authors, without undue reservation, to any qualified researcher.

## Abbreviations

GWI: Gulf War Illness
GW: Gulf War
TG: Triglycerides
DG: Diglycerides
CE: Cholesterol esters
PL: Phospholipids
SFA: Saturated Fatty Acids
PUFA: Polyunsaturated fatty acids
DHA: Docosahexaenoic acid
AA: Arachidonic acid
ME/CFS: Myalgic encephalomyelitis/chronic fatigue syndrome
RNC: Roskamp Neurology Clinic
GWIC: Gulf War Illness Consortium
CDC: Center for Disease Control
LC–MS: Liquid chromatography–mass spectrometry
ESI: Electrospray ionization
MS: Mass spectrometry
PC: Phosphatidylcholine
PE: Phosphatidylethanolamine
SM: Sphingomyelin
Cer: Ceramides
HexCer: Hexosylceramides
QC: Quality control
CV: Coefficients of Variance
MUFA: Monounsaturated fatty acids
ANOVA: Analysis of variance
MLM: Mixed linear modeling
B–H: Benjamini–Hochberg
SD: Standard deviation
VLDL: Very low-density lipoproteins
LDL: Low-density lipoproteins
GOLDN: Genetics of Lipid-Lowering Drugs and Diet Network
BMI: Body mass Index

## Declarations

### Disclaimer

The contents of this poster do not represent the views of the Department of Veterans Affairs or the United States Government.
