# Supplementary figures and images for: Sex-specific differences in plasma lipid profiles are associated with Gulf War Illness
Source: J Transl Med. 2022 Feb 5;20:73. doi: 10.1186/s12967-022-03272-3 (PMC8817550; doi:10.1186/s12967-022-03272-3)

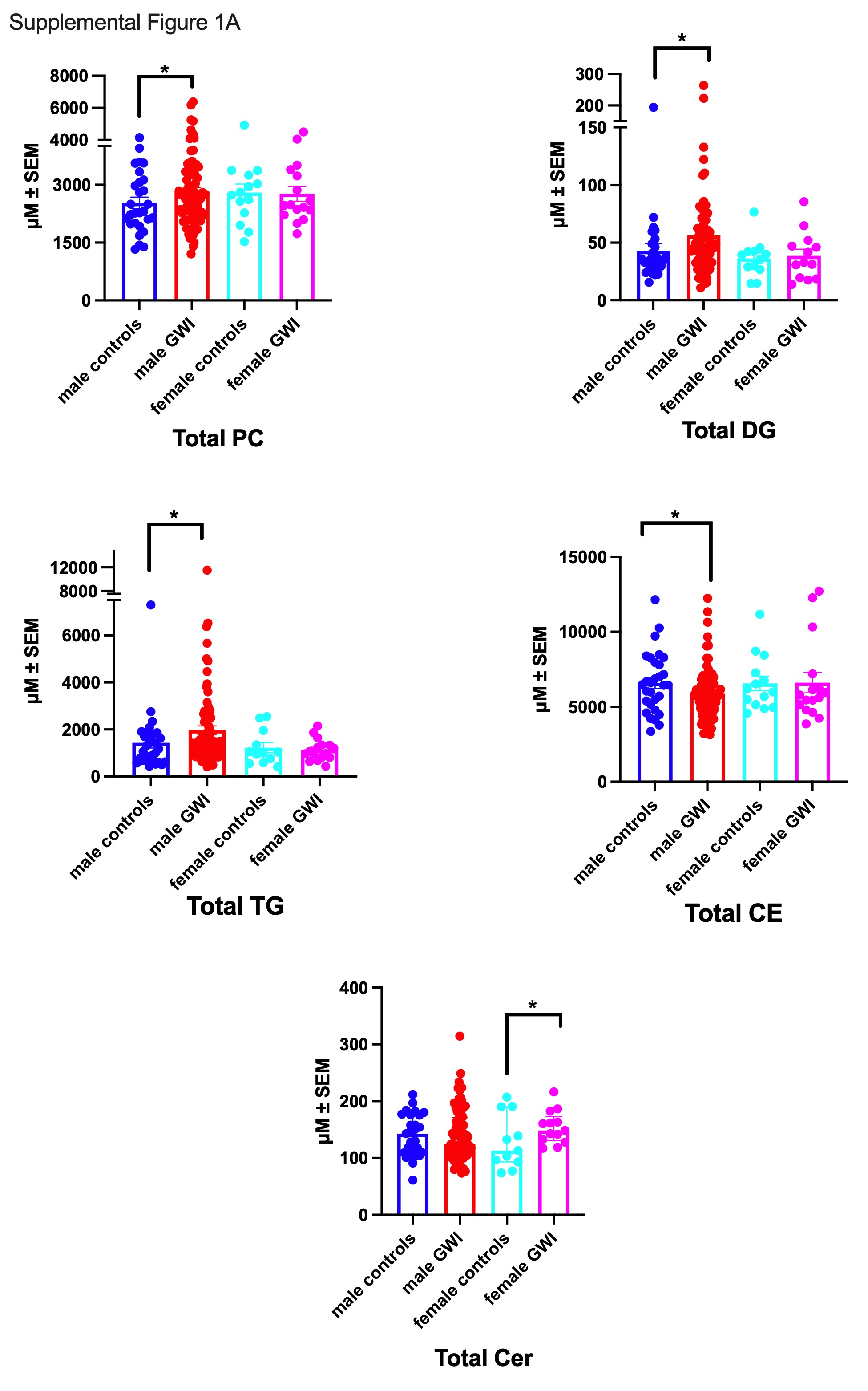

Supplement: Supplementary file 1 — Additional file 1: Figure S1. Individual levels differences in major blood classes between GWI and controls. Bar graphs indicate mean ± SE and individual values of total lipids (A), degree of unsaturation of each lipid class (B) and AA and DHA content of these lipids (C) that were significantly different between controls and GWI. Black asterisks (*) indicate significant differences between GWI cases and their sex-matched controls (p < 0.05). [file 12967_2022_3272_MOESM1_ESM.zip › Figure S1a.jpg]

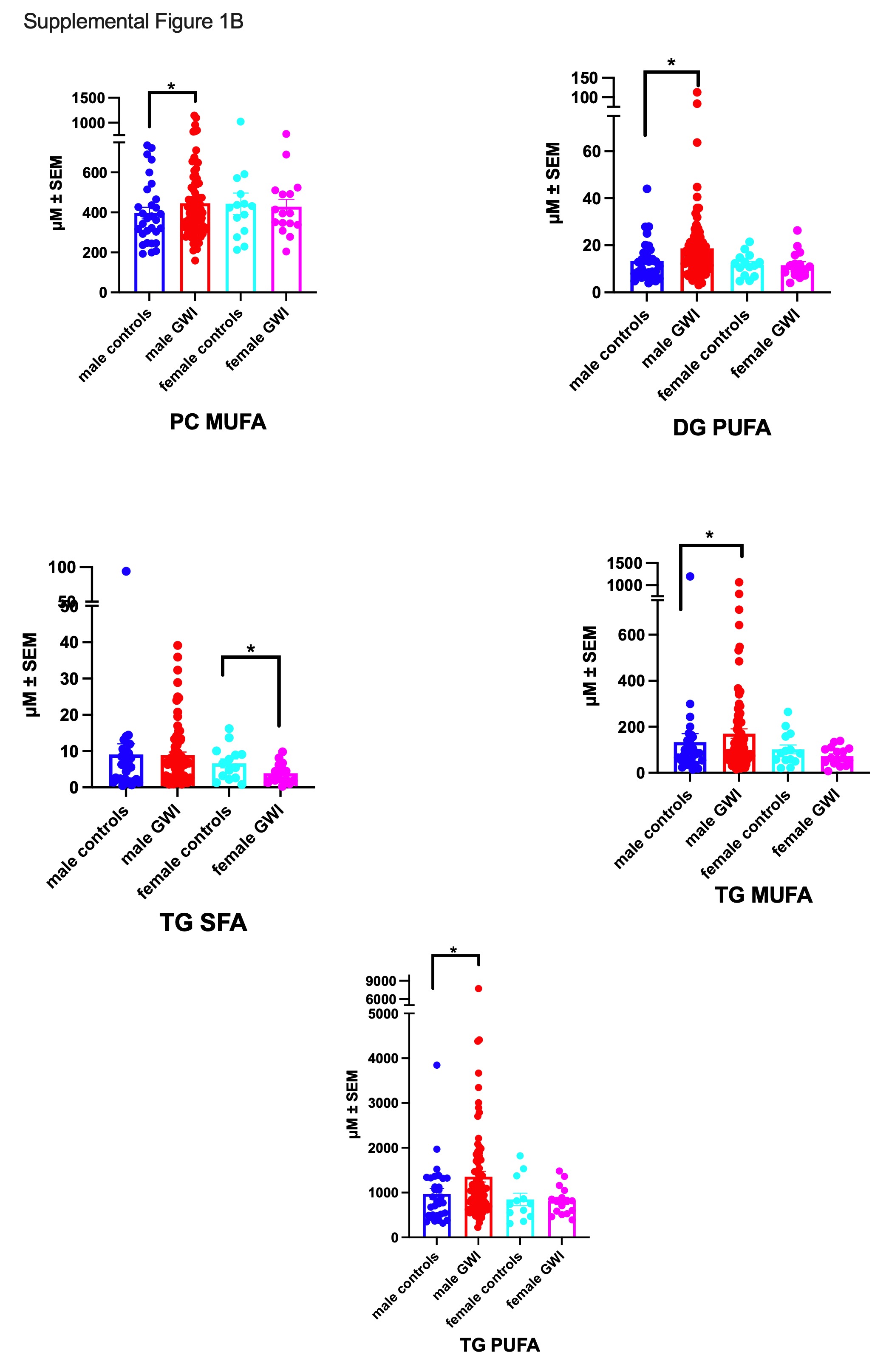

Supplement: Supplementary file 1 — Additional file 1: Figure S1. Individual levels differences in major blood classes between GWI and controls. Bar graphs indicate mean ± SE and individual values of total lipids (A), degree of unsaturation of each lipid class (B) and AA and DHA content of these lipids (C) that were significantly different between controls and GWI. Black asterisks (*) indicate significant differences between GWI cases and their sex-matched controls (p < 0.05). [file 12967_2022_3272_MOESM1_ESM.zip › Figure S1b.jpg]

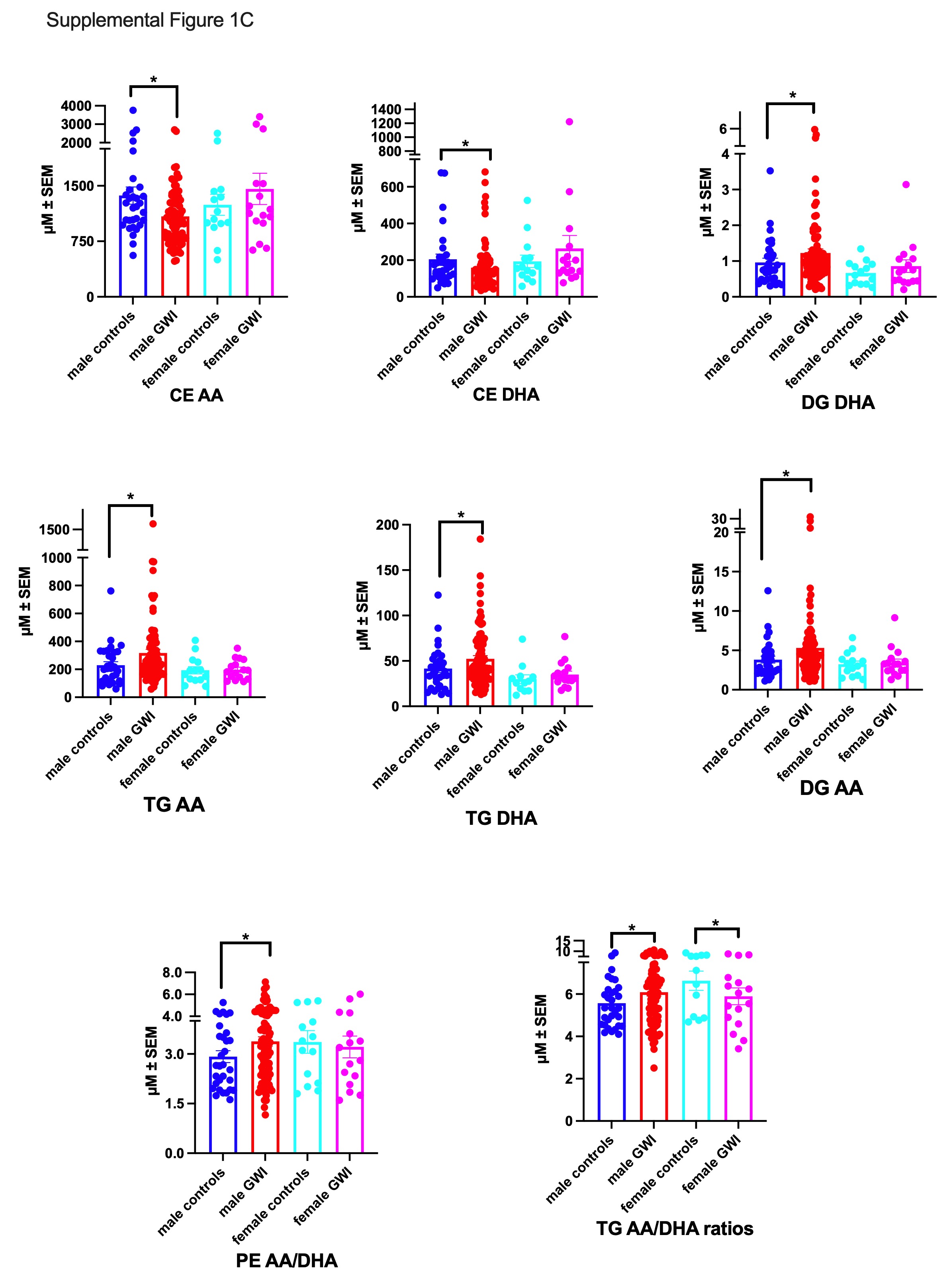

Supplement: Supplementary file 1 — Additional file 1: Figure S1. Individual levels differences in major blood classes between GWI and controls. Bar graphs indicate mean ± SE and individual values of total lipids (A), degree of unsaturation of each lipid class (B) and AA and DHA content of these lipids (C) that were significantly different between controls and GWI. Black asterisks (*) indicate significant differences between GWI cases and their sex-matched controls (p < 0.05). [file 12967_2022_3272_MOESM1_ESM.zip › Figure S1c.jpg]

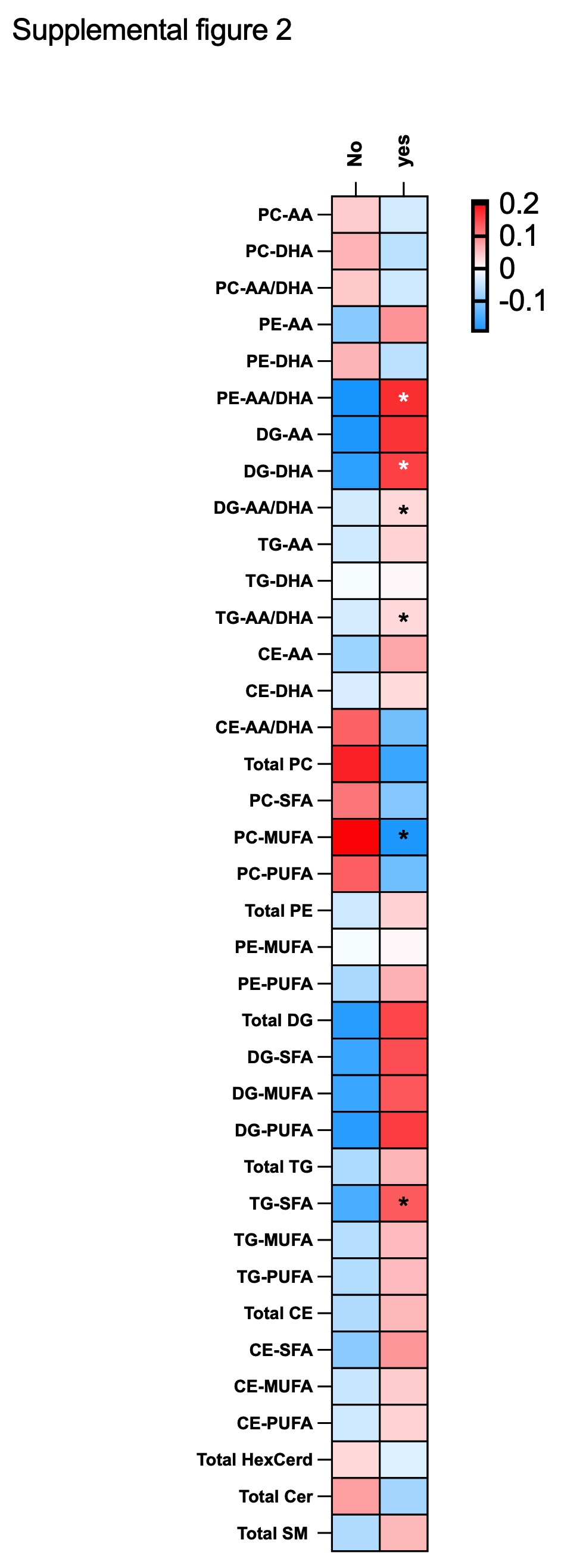

Supplement: Supplementary file 2 — Additional file 2: Figure S2. Association of blood lipid profiles and the presence of cardiovascular risk factors among the study population. Major lipids were further evaluated to determine the impact of presence of reported cardiovascular risk factors (no = 8 and yes = 32). Heatmap generated from z-scores transformed from individual concentrations. Differences in AA and DHA containing PL and neutral lipids were affected the presence of cardiovascular risk factors. Total lipid content was unaffected by cardiovascular risk factors. Black asterisks (*) indicate significant differences between GWI cases and their sex-matched controls (p < 0.05). [file 12967_2022_3272_MOESM2_ESM.jpg]
